# Development of Novel Unfolding Film System of Itopride Hydrochloride Using Box-Behnken Design—A Gastro Retentive Approach

**DOI:** 10.3390/ph15080981

**Published:** 2022-08-10

**Authors:** Shaima Alaithan, Nimbagal Raghavendra Naveen, Prakash S. Goudanavar, Penmetsa Durga Bhavani, Beveenahalli Ramesh, Naga Prashant Koppuravuri, Santosh Fattepur, Nagaraja Sreeharsha, Anroop B. Nair, Bandar E. Aldhubiab, Pottathil Shinu, Rashed M. Almuqbil

**Affiliations:** 1 Department of Pharmaceutical Sciences, College of Clinical Pharmacy, King Faisal University, Al-Ahsa 31982, Saudi Arabia; 2 Sri Adichunchanagiri College of Pharmacy, Adichunchanagiri University, B.G. Nagar 571448, Karnataka, India; 3 Department of Pharmaceutics, Vishnu Institute of Pharmaceutical Education and Research, Narsapur Medak 502313, Telangana, India; 4 School of Pharmacy, Management and Science University, Seksyen 13, Shah Alam 40100, Malaysia; 5 Department of Pharmaceutics, Vidya Siri College of Pharmacy, Off Sarjapura Road, Bangalore 560035, Karnataka, India; 6 Department of Biomedical Sciences, College of Clinical Pharmacy, King Faisal University, Al-Ahsa 31982, Saudi Arabia

**Keywords:** itopride hydrochloride, gastro retentive systems, unfolding film, Box-Behnken design, optimization

## Abstract

Currently, gastro-retentive dosage forms achieved a remarkable position among the oral drug delivery systems. This is a broadly used technique to hold the drug delivery systems for a long duration in the gastro intestine (GI) region, slow drug delivery, and overcome other challenges related to typical oral delivery such as low bioavailability. The current work aimed to formulate and characterize a new expandable gastro-retentive system through Itopride Hydrochloride (IH)’s unfolding process for controlled release. The IH-loaded unfolding film formulation was optimized using the Box-Behnken design for folding endurance and length of tested layer (LTL). Initially, the formulation was made using several anti-adhesive additives to promote the unfolding mechanism. Citric acid and sodium bicarbonate were selected as anti-adhesives based on these results. The enfolded film in a capsule shell was shown to unroll in the stomach fluids and render drug delivery up to 12 h in acidic conditions. A fabricated system should have dimensions more than the size of the relaxed pyloric sphincter, and as required, >20 mm LTL was identified. This further confirms that the residence period in the stomach is irrelevant to the fed or fasted condition. Based on desirability criteria, the formulation containing 143.83, 0.7982, and 14.6096 Eudragit L100, PEG, and sodium bicarbonate are selected as optimized formulations (O-IH-UF). The optimized formulation was further analyzed for various parameters such as tensile strength, mechanical strength, unfolding nature, degradability, and in vitro release studies. The pharmacokinetic study revealed greater AUC (area under the curve) and long half-life with the designed O-IH-UF formulation, confirming that the unfolding film type can be a favorable drug system for enhancing the bioavailability of low soluble drugs. The results showed that unfolding types of gastro retentive systems could potentiate the drugs with stability issues in an alkaline medium or those with absorption in acidic conditions.

## 1. Introduction

The oral route is a promising path for drug delivery as it can be flexibly formulated, administered efficiently, and maintain patient compliance [1]. However, in humans, emptying time via significant absorptive portions of the stomach and intestines is usually around 2–3 h, resulting in improper drug release and decreased drug efficacy [2]. The retention period of the controlled oral drug system is consistently below 12 h [3]. The latest patent and scientific literature showed an increasing trend in new drug systems held in the GI region for a predictable and extended duration. Currently, various approaches such as floating, bioadhesive, swelling, expandable systems, and slow emptying devices are used to retain dosage systems in the gastric region. The distinctive nature of these systems is related to the various advantages attained by formulating them. The advantages of these systems are improved bioavailability, minimal side effects, less dosing frequency, and good patient compliance [4,5,6].

The retention of the conventional drug systems is affected mainly by two factors, (i) fed state or fasted condition and (ii) the size of the formulation. Suppose the dimensions of the drug system are more than the size of the relaxed pyloric sphincter. The system will have a long residence period in the stomach irrespective of fed or fasted condition. Expandable systems are one among them that will expand on contact with stomach fluids after being folded in the capsule. This enlargement makes the system dimensions greater than the relaxed pyloric sphincter (12.8 ± 7 mm), providing mechanical support and thus avoiding system eviction [5,7]. The suitability of hard-shell gelatin capsules is a benefit for such dosage forms. To alter drug release by using polymeric films, there are various concerns, such as choosing a polymer with the required capabilities to unwind and enlarge in the gastric system and complex processes in preparing polymeric films packed with a drug [8]. A distinct approach to enhancing the gastric residence period is the floating system’s integration, which can enlarge by unrolling and bulging with degradable polymers (hydrophilic/phobic).

Gastro retentive systems are employed for drugs with a narrow absorption range, drugs requiring multiple dosing, and drugs with a half-life around 2–8 h [9,10]. Itopride Hydrochloride (IH) is a gastro kinetic drug indicated for the use of dyspepsias and other gastric diseases such as anorexia, fullness, pain in the upper quadrant of the abdomen, and functional dyspepsia [11]. It has a narrow absorption window and short biological T_1/2_ of 5–6 h with the upper GIT as a primary site of absorption, which results in insufficient absorption, quick clearance, and sub-optimal drug concentrations in the blood [12,13]. Additionally, the presence of food has no impact on the bioavailability of IH. Due to the incapacity of the typical control released preparations to achieve the required drug release profile within the absorption window, the gastro retentive of IH was designed for sustained drug delivery with enhanced therapeutic effectiveness [14].

Despite their interesting characteristics, expandable systems have few challenges. Storage of such easily hydrolyzable, biodegradable polymers is difficult. For the unfolding systems, the mechanical shape memory is relatively short-lived [15]. Finally, expandable systems should not interfere with gastric motility, must be readily biodegradable, and must not have sharp edges or cause local damage on prolonged retention. Permanent retention of rigid, large single-unit dosage forms may cause bowel obstruction, intestinal adhesion, and gastropathy [16]. In connection to this, the polymeric films loaded with the drug were examined formerly, and the influence of folding manner, shape, and polymer features on gut retention was studied. Klausner et al. conducted the first study on Riboflavin and Levodopa expandable gastro retentive dosage forms [17]. Later on, though many research works were carried out on expandable systems, special attention needed to be given to work done by Alice Melocchi et al. in developing shape memory polymers through 4D printing and extrusion. They made prototypes containing allopurinol, directly manufactured by fused deposition modeling or shaped by purposely-designed templates from hot-melt extruded rods immediately after production [18]. Levetiracetam expandable gastro-retentive dosage form, based on an unfolding mechanism, was optimized using a simple lattice design considering the concentration of hydroxy propyl methyl cellulose, Carbopol 934P, and xanthan gum as independent variables [19]. A few notable research works have been identified as developing unfolding-type expandable gastro retentive film of enalapril maleate [7] and unfolding-type gastro retentive film of Cinnarizine based on ethylcellulose and hydroxypropylmethylcellulose [8]. 

## 2. Materials and Methods

### 2.1. Materials

Wallace Pharma, Goa, India, presented IH. Eudragit L100 and poloxamer P407 were procured from Otto Chemie Pvt Ltd., India. Gelatin, ethanol, citric acid, and sodium bicarbonate were procured from Yarrow Chemicals. The other chemicals and reagents used were of an analytical grade type and obtained from manufacturers.

### 2.2. Fourier Transform Infrared Spectroscopy (FTIR)

The FTIR spectral readings were taken at room conditions with an IR spectrophotometer (Perkin Elmer Instruments, North Billerica, MA, USA). This was performed to evaluate the peak patterns and for comparative use [20]. The resolution was kept as 4 cm^−1^, as it indicates the degree of fitness of the data obtained by measurement. The spectral readings of IH and the physical mixture of IH were recorded at a wavelength of 400–4000 cm^−1^ with a KBr disc [21].

### 2.3. Preparation of IH loaded Unfolding Film (IH-UF)

IH-UF was formulated using the solvent evaporation method. Absolute ethyl alcohol (5 mL) was added to a beaker and placed on a hot plate. Other ingredients were added gradually and mixed with aquaphobic polymers that were hard to dissolve. Further, Eudragit L100 and 2.5 mL of the solvent were added, and subsequently, gelatin, IH, PEG, and poloxamer P407 were added [5]. Constant stirring was performed to obtain a homogeneous mixture. The resultant mix was then shifted to a glass mold, kept in an oven, and subjected to heating at 70 °C for 120 min to evaporate the solvent and solidify. Before adding the mixture, an anti-adherent plastic bag was placed in the glass mold to easily remove the dry layer. The obtained layer was placed in a desiccator for 48 h and later sliced to the required dimensions (20 × 30 mm). It was weighed, folded into an appropriate shape by hand, and kept in a “00” sized hard-shell gelatin capsule [Sunil Health Care, India]. 

### 2.4. Selection of Anti-Adhesives for the Unfolding of IH-Loaded Film

The purpose of anti-adhesive agents (microcrystalline cellulose, starch, talc, magnesium stearate, talc, sodium bicarbonate, and citric acid) is to prevent the prepared layers’ stickiness and aid in the unfolding process. A rightful unfolding test can be achieved when the test layers unfold to a minimum of 18–20 mm in 10 min. The test was conducted in a USP dissolution type II apparatus containing hydrochloric acid (1.2 pH) medium. Following the 10 min after testing, layers were taken, and their length was measured. Consequently, based on these results, an ideal agent was chosen as an anti-adhesive excipient.

### 2.5. Experimental Design 

The preparation of IH-UF was standardized by the statistical model RSM (Response Surface Methodology). The concentrations of Eudragit L 100 (*X*_1_), PEG (*X*_2_), and sodium bicarbonate (*X*_3_) were chosen as individual parameters at three distinct levels encoded as −1 (low), 0 (medium), and +1 (high). Sodium bicarbonate was chosen as one of the variables to study its impact on the gastro retentive nature of the film. PEG imparts flexibility to the film, which is considered the most vital parameter to folding the final formulated film into the capsule shell and expanding the film. Eudragit is also considered in the design to study its impact on the folding endurance and also to owe to its drug release retarding nature. All these parameters were scrutinized for their influence on folding endurance and length of tested layer (LTL) using the Box-Behnken model of Design Expert 12 (Stat Ease Inc., USA), originating 17 experiment runs [22,23]. Table 1 represents the total experiment plan, coded and actual values of chosen parameters, and restraints of the chosen responses. Analysis of variance (ANOVA) validated the developed polynomial equations. Furthermore, various statistical tools were employed in all test runs to select the best fit model. A quadratic design was employed in every test run to quantify the outcome response and regression analysis.

$$Y_{i\left(Quadratic\right)}=b_{0}+b_{1}X_{1}+b_{2}X_{2}+b_{3}X_{3}+b_{4}X_{1}X_{2}+b_{5}X_{1}X_{3}+b_{6}X_{2}X_{3}+b_{7}X_{1}^{2}+b_{8}X_{2}^{2}+b_{9}X_{3}^{2}$$

where,

*Y_i_*—chosen response or dependent variable;

*B_o_*—computed response;

*b_i_*—estimated coefficient for main effects (*X*_1_, *X*_2_*, X*_3_), interaction terms of main effects (*X_1_X_2_*, *X_2_X_3_*, *X_1_X_3_*), and polynomial terms of independent variables (*X*_1_^2^, *X*_2_^2^, *X*_3_^2^). 

#### 2.5.1. Folding Endurance 

This test was conducted by folding the developed films continuously in one manner at the same spot until the film gets shattered (angle, 0–90°; speed, 30 folds per min). Endurance value is the frequency of folding in the film at the same spot without breaking it. This gives the indirect measure of film rigidity; that is, a lesser endurance value denotes the fragile nature of the film [24,25].

#### 2.5.2. In Vitro Unfolding Test and LTL

The developed polymer film was pleated in two distinct manners (rolling and accordion) and placed in 00 capsules. In vitro unfold test was performed in USP type I apparatus (TDT-08 L, Electrolab, Mumbai, India) consisting of 900 mL of 0.1 N HCL (pH 1.2) as a dissolving medium kept at 37 ± 0.5 °C temperature with 50 rpm speed. To assess the unfolding nature, baskets were taken out, and after 10 and 15 min, the layers were removed, and their length was measured. 

### 2.6. Optimization and Validation of Standardized Outcome

Design-Expert software was used to generate responses given by all the formulations. The study methodology was designed through the obtained responses, and also a response surface graph was plotted. A numerical standardization method was used to develop a standard formula with each variable’s specific upper and lower limits. The obtained results were combined into a desirability function [26]. The outcomes group was divided with the higher desirability, and the best outcomes that meet the specifications were noted. The response surface plot was used to establish the association of dependent and non-dependent parameters. ANOVA analyzed the effect of different factors on coefficients of the slope. Furthermore, the relative uncertainty was calculated based on the variance of predicted and experimental values.

### 2.7. Evaluation of Optimized Formulation

#### 2.7.1. Weight Variation

This test was performed for ten patches (1 × 1 cm). Individual weight was noted for all the patches. Further, the mean weights and standard deviations were computed [27]. The mean ± SD values (*n* = 3) are mentioned in Table 2.

#### 2.7.2. Thickness

An optical microscope was used to estimate the thickness of the patch (DeXel-FilTrate Optical, Conation Technologies, Pune, Maharashtra, India). Transverse sections were taken from various points within the patch and observed under a microscope with 100× magnification. The average and standard deviation were computed [28].

#### 2.7.3. Tensile Strength

Tensile strength is the proportion of the greatest load the sample film can bear without breaking when elongated to the initial area of the cross-section of the sample. The weights were raised slowly to augment the pulley power until the test film became fragile. Using a magnifying glass on a graph sheet, the percentage of elongation before the fracture of the film was noted, and tensile strength was calculated [29] (V TECH Digital, Nunes Instruments, Coimbatore, India).

#### 2.7.4. Mechanical Strength

Mechanical strength is the ability to resist the exerted manual force. A bursting paper machine was used for the test. The machine exerts mechanical stress on a ball that progresses through the sample film when positioned horizontally in a plane after rupturing it [30].

#### 2.7.5. Degradability Test

A degradation test was carried out to determine the degradation capacity of the standard formula at intestinal pH with a simulated buffer solution of potassium phosphate (pH 6.5). Before loading the capsule, the layer was kept in the medium directly [7]. Its rigidness must be reduced under alkaline conditions. A Young’s modulus test was conducted manually to analyze the rigidity. The results enable us to forecast layer nature in the intestines if preterm eviction occurs.

#### 2.7.6. Scanning Electron Microscopic (SEM) Studies

SEM was used to examine the polymeric film’s morphology. The film was analyzed with a JSM-6490LA electron microscope employing secondary electron imaging at 5 kV acceleration voltage at 3493 and (b) 6987 magnifications.

### 2.8. Drug Content

The patches, equal to 0.05 g of IH, were placed in an HCL (100 mL of 0.1 N) solution of pH 1.2 to extract the drug for about 6 h. The resultant solution was strained and examined through UV absorption spectroscopy at 258 nm (Shimadzu UV-1700, Shimadzu Corporation, Kyoto, Japan) [31]. 

### 2.9. Gastric Retention Time and In Vitro Dissolution Studies

In vitro test was carried out in USP apparatus II with 500 mL hydrochloric acid medium (pH 1.2), resembling the low stomach pH. Samples were introduced at 0, 1, 2, 3, 4, 6, 8, 10, 12 and 14 h [32]. Sink conditions were maintained; 2.5 mL of sample was drawn out for analysis. The test was repeated with acetate buffer (pH 4.1) as a medium that resembles high stomach pH [33]. While developing the layers for the dissolution test, paper clamps were utilized to support the test layers to the bucket base. To study the release mechanism from the optimized formulation, three kinetic models were considered to fit the experimental data. Zero order Q = Q_0_ − K_0_t (Q is the amount of drug released at time t, Q_0_ is the amount of drug remaining in the formulation, K_0_ is zero-order release rate constant); Higuchi model, Q = K_2_t_1/2_ K_2_ is Higuchi rate constant; Peppas model, Q/Q_0_ = K t*_n_* (Q/Q_0_ is a fraction of drug release at time t, K is a constant, and *n* is the diffusion exponent indicating the mechanism of drug release).

### 2.10. Stability Studies

The stability study was performed for a short duration of the optimized formulation IH-UF. Required films were kept in amber-colored bottles and securely closed with a rubber stopper. The glass bottles were preserved in stability chambers for six months at 40 °C ± 2 °C + 75% RH ± 5% RH and 25 °C ± 2 °C + 60% RH ± 5% RH. Samples were collected at various periods and analyzed for endurance, unfolding behavior, and drug release patterns. The in vitro study results were compared with the zero-time drug release pattern through similarity factor (f_2_) to find the variance [34].

### 2.11. In Vivo Evaluation Studies

All the in vivo studies were approved by the Institutional Ethical Committee of India (SACCP-IAEC/2021-01/31). 

#### 2.11.1. Buoyancy or Gastric Residence Studies in Rabbits by Using X-ray Imaging

By replacing IH and modifying the additive amounts, barium sulfate (10%) was added to the formulation to make the dosage X-ray visible. Male albino rabbits weighing 2.5 to 3 kg were used for this, and the animals were fasted for roughly 12 h before the procedure. An initial abdominal X-ray was performed to check that there were no radio-opaque materials present. O-IH-UF was delivered with the help of an oral cannula and 15 mL of water. Throughout the course of the trial, 10 mL of water was supplied every interval (up to 6 h). Under the direction of a radiologist, the animal was held upright while X-ray images of its abdomen were taken [35]. 

#### 2.11.2. Pharmacokinetic Studies

We used six male New Zealand white rabbits (*n* = 12), each weighing 3.08 kg ± 0.11 kg. They were fed a commercial laboratory rabbit diet, and each was housed in a stainless-steel cage. The rabbits had unlimited access to water while fasting for 12 h before and during the pharmacokinetic trial [36]. The tests were conducted on conscious animals the entire time. A dose of IH equal to 2.5 mg/kg was administered to all the animals. The intended dose of the commercial tablets (Ganaton 50 mg, Abbott Laboratories, India) was given to Group I (*n* = 6). An unfolding film containing 2.5 mg/kg IH was folded into a capsule and given to Group II (*n* = 6).

Following the delivery of both samples, a blood sample (1.5 mL) was taken from the rabbits’ marginal ear vein at intervals of 0.25, 0.5, 1, 2, 4, 6, 8, 10, and 24 h. To prevent clotting, blood samples were drawn into EDTA tubes, and to get the plasma, the tubes were spun at 4000 rpm for 15 min. Before further investigation, the separated plasma tubes were kept in a −20 °C freezer. The HPLC system (Agilent 1260) with a UV detector was employed as the chromatographic system. According to Yehia et al., all samples were analyzed at room temperature using the HiQsil C18 column (25 cm) and Levofloxacin as an internal standard [14,33,34].

A volume of 225 µL of rabbit plasma was taken out, and 25 µL of a standard levofloxacin solution was added as an internal standard (IS), bringing the total amount to 250 µL. The solution was thoroughly mixed after each stage, followed by the addition of 4 mL of dichloromethane, a 3 min vortex, and 5 min centrifugation at 5 °C and 5000 rpm (VWR VV3 S540 International West Charter; California, USA). A vacuum oven (VACUCELL VUS-B2V-M/VU 22, MMM Group; Ettlingen, Germany) was used to evaporate the supernatant after it had been decanted. The residue was first dried, then reconstituted with 200 µL of mobile phase, and injected into an Agilent HPLC Agilent 1260 for analysis [37].

##### Analysis of Pharmacokinetic Parameters

Plasma concentration vs. time data were controlled, and the pharmacokinetic characteristics of the EH test were estimated using a non-compartmental technique. PK Solver calculated the data. Microsoft Excel’s free menu-driven add-in software PK Solver was created in Visual Basic for Applications (VBA) [38].

## 3. Results and Discussion

### 3.1. FTIR

**Figure 1:**
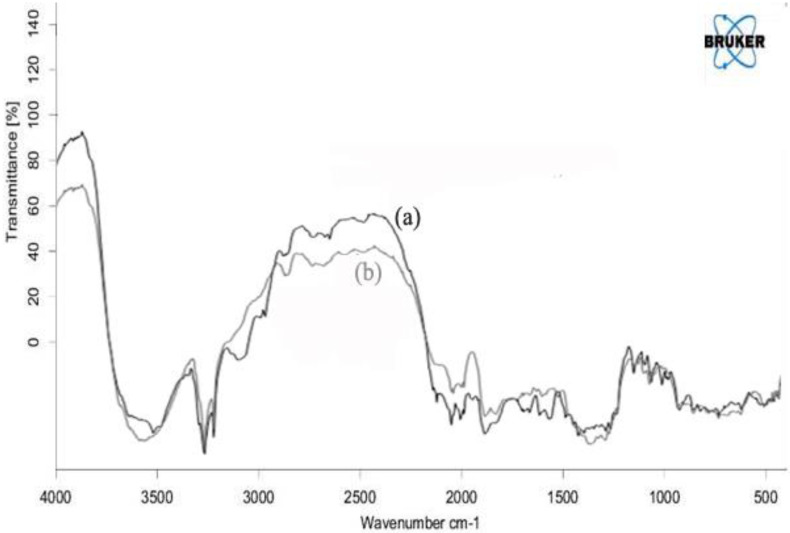
FTIR spectra of (a) IH and (b) physical mixture of IH + excipients at a wavelength of 400–4000 cm^−1^.

FTIR spectra were recorded for IH and the physical mixture of IH with all the additives (Figure 1). The spectra of IH exhibited peaks as 1267.97 cm^−1^ for C-O-C asymmetrical ether (alkyl) stretching, 1028.84 cm^−1^ for C-O-C asymmetrical ether (aryl) stretching, 3281.29 cm^−1^ and 3226.33 cm^−1^ for NH stretch, 1631.48 cm^−1^ for NH bending, 1651.73 cm^−1^ for C=O stretching, 1147.44 cm^−1^ for C-N stretching, and 2942.84 cm^−1^ and 2965.02 cm^−1^ for C-H. The spectra of the physical mixture exhibited the majority peaks of IH at 1270 cm^−1^, 1020 cm^−1^, and 1149 cm^−1^. Hence, the combined FTIR spectra of the physical mixture of drug and polymers did not significantly shift the intensity of IH peaks, denoting that there were no interactions with other excipients used in the preparation. 

### 3.2. Selection of Anti-Adhesive Additives and Optimization of IH-UF 

**Figure 2:**
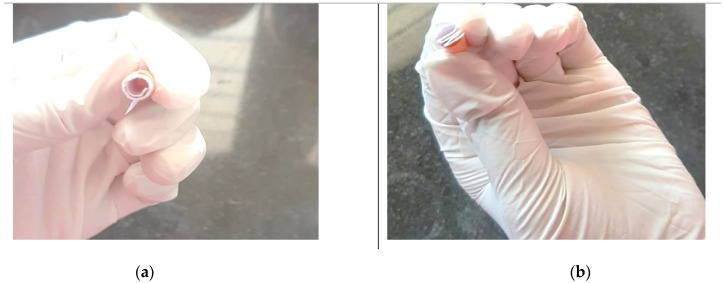
(**a**) Rolling and the (**b**) accordion pattern of folding for IH film to study the impact of the folding method on the unfolding of IG gastro retentive film.

A decrease in mechanical shape resulting from the force exerted is a significant obstacle for unfolding the films. Polymer substances having glass transition temperatures near room temperature with the flexibility to retain their actual structure are helpful in this type of preparation. They go through slight distortion and regain their elasticity as needed [18,19]. The unfolding nature of the film’s roll and the accordion pattern (Figure 2) was not acceptable as they were noted to be sticky with each other. After coating it with anti-adhesive materials, accordion folding unfolds avidly compared to roll folding. The unfolding nature of the film was tested using different anti-adhesive materials, and the results are presented in Table 2. 

The anti-adhesives are used to avoid the stickiness of the prepared layers’ excipients and aid unfolding. Citric acid and sodium bicarbonate were made into fine powders and dispersed on the formerly developed layers, considering the anti-adhesives employed for the unfolding test. The test was completed in a USP II apparatus with HCl (pH 1.2) medium. The sample layers were taken out after 10 and 15 min, and length was measured. Microcrystalline cellulose, magnesium stearate, and talc did not provide any unfolding nature to the film. A good unfolding test is attained when the test layers unfold to a minimum of 18 mm within 15 min. 

As required, citric acid and sodium bicarbonate were studied on three other samples that rendered good results with lengths greater than 18 mm. This combination liberates carbon dioxide that drives the folded layers to get away from each other. Hence, this combination is regarded as the best anti-adhesive excipient and is considered one of the variables to optimize the formulation. The desired ratio between sodium bicarbonate and citric acid needs to have superior unfolding nature. Increased citric acid ratio results in aggregates when blending with sodium bicarbonate, ultimately reducing the stickiness of the resultant powder towards the developed layers.

The Box-Behnken design of RSM was used to estimate the standard level of the chosen factors and their interactivity in obtaining the preferable folding endurance and LTL. Overall, 17 experimental runs were completed, and the obtained responses are shown in Table 3. The folding endurance of all study preparations was between 69 and 114, while the length (LTL) was in the range of 9 to 25 mm. All the developed patches had a folding endurance greater than 69, denoting good integrity and flexibility. It was noted that PEG 400 is crucial for this delivery system, without which the dosage form becomes fragile. The obtained results were scrutinized for individual responses and the effect of parameters through statistical analysis by using fx and ANOVA. A quadratic model was adopted for both responses based on the sum of squares, model summary, and fit summary (Table 4 and Table 5). A high-degree quadratic polynomial was selected, where the auxiliary terms can be noted, and the model is not aliased.

For both responses, the Predicted R^2^ values of 0.8997 and 0.8762 were in rapport with the Adjusted R^2^ of 0.9821 and 0.9756 correspondingly, as the disparity is below 0.2. Apart from this fit, summary data were used to confirm the fitness and effectiveness of the selected model. The reproducibility of the model can be ensured with the value of the coefficient of variation. The CV value of below 10% confirms the model repeatability. Comparatively fewer CV values (1.87 folding endurance and 4.37 LTL) were observed during the test, indicating the accuracy and reliability of the selected model. Adequate precision quantifies the quotient of signal to noise. Usually, a fraction > 4 is considerable. Folding endurance and LTL showed this ratio of 34.9242 and 28.5860, indicating a suitable signal. Hence, ensuring the model efficiency in operating the design space. Lack of fit can give an ineffective model that interprets actual data. Thus, lack of fit is essential to confirm that the equations generated by the model are reasonable in predicting the responses. The responses’ *p*-values corresponding to lack of fit were not significant, so the selected model was suitable for the study [39]. The F-values of both responses were 98.74 and 72.17, interpreting the model applicability. There was only a 0.01% likelihood that a high F-value was due to noise, and the *p*-value of the model was found to be significant (<0.0001). 

**Figure 3:**
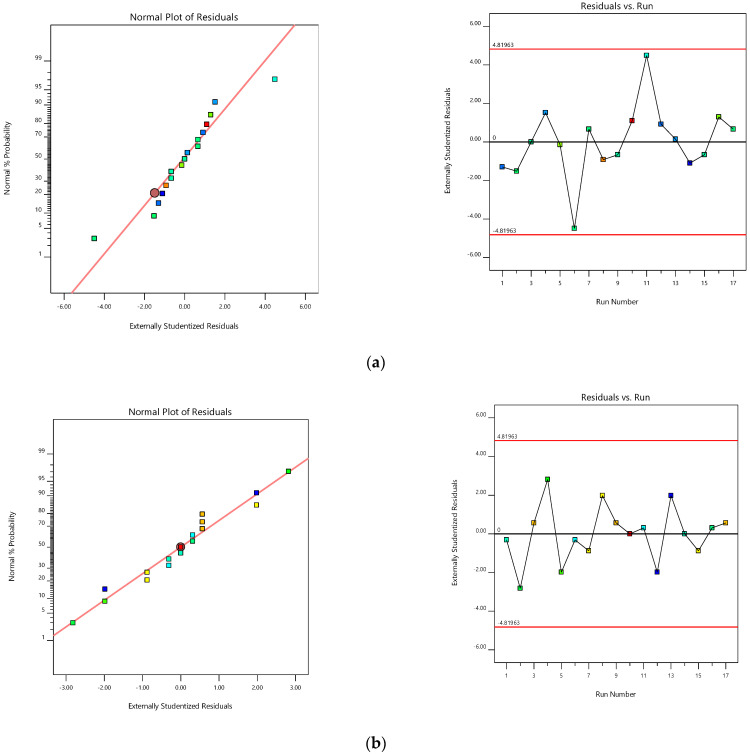
Normal probability and model residuals versus test orders for (**a**) folding endurance and (**b**) LTL.

A huge interrelationship was observed between the experimental and predicted values while indicating the chosen responses. The probability distribution reveals that residuals are below the normal distribution (straight linearity). The standard statistical tools are not used, while inspection of the visible plot is suitable. Furthermore, a usual residual graph (external studentized residuals vs. usual probability percent) was plotted to quantify and assure model accuracy [40] (Figure 3a). Furthermore, the influence of test orders on the model was demonstrated by the residuals against test order [41]. In the current study, a slight deviation was noted in the linear distribution of the external studentized residuals inferring that the chosen model was statistically acceptable [42]. Figure 3b represents the experimental run operated against the residuals, which is a working process to identify the slinking variables that can modify the test results. A random distribution fashion was observed in the chart, indicating the lurking of time-dependent parameters in the framework.

The root effect interconnection of the chosen variables and the individual responses can be exemplified by the suggested quadratic polynomials and the respective statistical significance estimated by using ANOVA. ANOVA was carried out to test the interference of measurable effects of the factors. Polynomial equations were obtained by applying multiple regression to the data. The equations generated from the output of the possible standard model are described below:

Final equation in terms of coded factors:

Folding endurance = +85.00 + 7.50 A +14.13 B + 3.63 C + 5.00 AB + 1.50 AC + 2.75 BC–0.6250 A^2^ + 2.12 B^2^ + 1.13 C^2^

LTL = +21.60 + 2.63 A + 2.88 B + 2.50 C + 1.50 AB + 0.2500 AC + 0.2500 BC − 2.55 A^2^ − 1.05 B^2^ − 5.80 C^2^

For both responses, ANOVA coefficients, together with *p*-values, are presented in Table 6. The results attained were used to determine the significance of model coefficients. ANOVA results outranged the significance level produced by the quadratic polynomials. Further, the *p*-value was <0.0500, indicating the significance of model terms. The test method specified that folding endurance was significantly affected by synergic effects of A, B, C, AB, BC, and polynomial terms of B with a *p*-value of <0.0001, <0.0001, 0.0004, 0.0004, 0.0113, and 0.0306 likewise. Response 2 was affected by, (i) adversary consequences of the polynomial term of A, B, and C with *p*-values 0.0002, 0.0241, and <0.0001 and (ii) synergistic output of A, B, C, AB (*p*-value < 0.0001) and AB (*p*-value of 0.0052), and among the critical parameters, term B affected both responses with great enormity, followed by factor A. 

**Figure 4:**
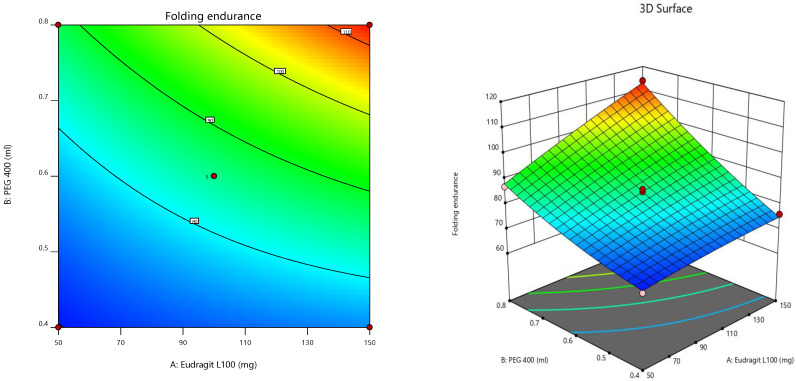

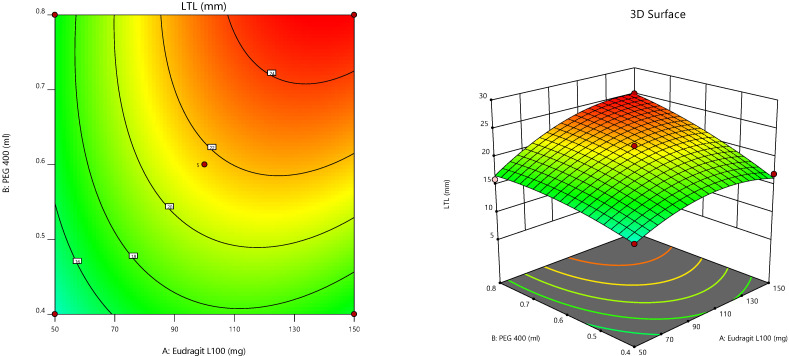
Contour plots and 3D Response surface plots for (**A**) Folding endurance and (**B**) LTL.

Furthermore, the consequences of independent parameters on responses were analyzed using RSM (Figure 4) [43,44,45]. The contour plot representing the relation of selected responses with the variables gives the variance effects. RSM was used to determine and elucidate the response of individual parameters over the different responses attained [46,47]. Three-dimensional surface graphs are essential to demonstrate the interactions and main effects. The responses attained are interpreted using contour/level plots [48].

**Figure 5:**
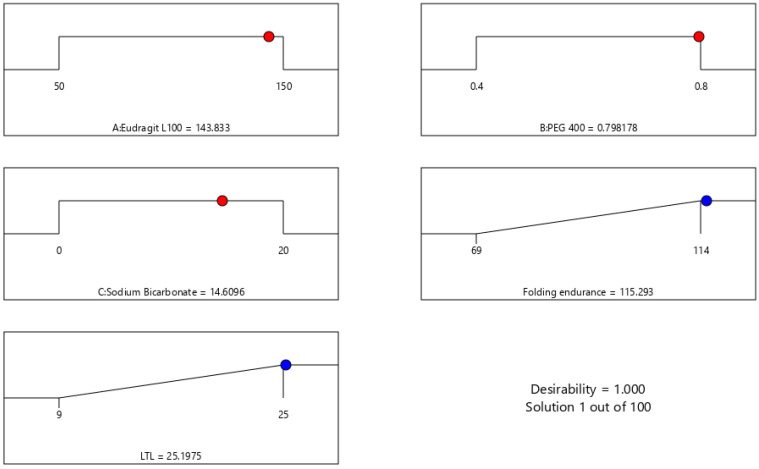
Desirability plot of selected BBD in optimizing the concentrations of chosen variables.

The global desirability function (D) was used to optimize the model’s order attained by statistical methods. Both responses were laid a maximum limit to acquire an inlay graph to strengthen the independent variables. All possible independent variables were considered in the method for optimization. In the desirability function plot, the individual parameters (optimal level) signified a maximum of 1.00 D value for both responses. The optimized concentrations of Eudragit L100, PEG, and sodium bicarbonate were 143.83, 0.7982, and 14.6096 (Figure 5). Hence, implementation of this system aids in obtaining a folding endurance of 115.29 and 25 of LTL. A standardized preparation [O-IH-UF] was formulated and evaluated to check the study design. The relative error was less than 5%, ensuring the accuracy of the design (Table 7). The same preparation was employed to determine other parameters. 

### 3.3. Evaluation of Optimized Formulation

O-IH-UF was evaluated for various physicochemical properties, and the result is summarized in Table 8. The uniformity of weight of the patches was observed in the range of 0.253 ± 0.023 g. The patches had a thickness of 0.93 ± 0.02 mm. For all the patches folding endurance was more than 100, denoting good flexibility and integrity.

Mechanical strength is the tangential force exerted on the ball through a uniform area in the film. A bursting machine was used for the test. Positive results were obtained, inferring that O-IH-UF has an adequate mechanical strength, which can be credited to the standardized concentrations of PEG 400 and Eudragit L 100.

**Figure 6:**
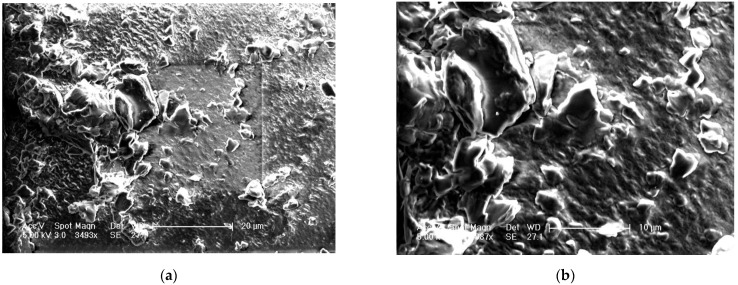
SEM image of optimized formulation at (**a**) 3493 and (**b**) 6987 magnification.

The surface morphology of the optimized film is shown in Figure 6a at 3493x and Figure 6b at 6987x. The surface morphology of the polymer film has shown particulate matter scattered on the surface with several depressions, which can be due to the effervescent nature of anti-adhesives used for the unfolding of the film. This can further make a channel for leaching of IH at the initial periods, which accounts for rapid drug release. The degradability test (pH 6.5) of the prepared layers illustrates improved rigidity and thickness in wet than dry conditions. Samples from standardized formula F.T were tested at pH 1.2 and 6.5 with type II apparatus. The Young’s modulus for the wet samples tested at pH 6.5 was 0.110 N/mm^2^ after 5 h, while samples tested at pH 1.2 were 0.302 N/mm^2^ after 6 h. The layers examined at pH 6.5 and 1.2 had a thickness of 1.24 mm and 2.02 mm, respectively. The results showed that the alteration in gastric pH due to food intake, disease, and drugs would not modify the drug release or enlarge the prepared drug system. It was noted that both rigidity and thickness were reduced significantly at intestinal pH (6.5) than at the stomach pH (1.2) [7]. These results denote that the formulated system disintegrated quickly, was more elastic at intestinal pH, and will not remain in the intestines for longer, leading to side effects if preterm eviction occurs.

**Figure 7:**
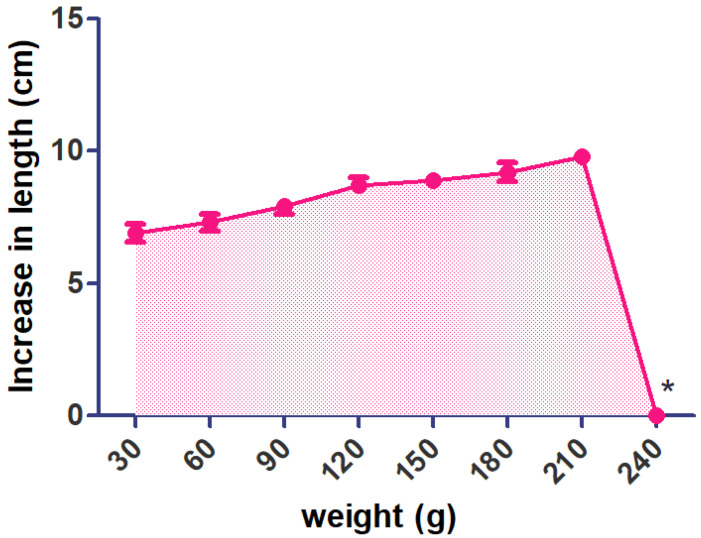
Effect of added weights on change in length of the film (Increase length of film observed concerning added weights. (*, film broken)).

A notable change was seen in the length of the optimized preparation by using 30–210 g of weight. These results indicate that PEG offers a good plasticizing effect for the polymeric film yet increases the penetrability of water vapor in the film. I-IH-UF have a maximum tensile strength of 240 g (breaking of the film was observed) (Figure 7).

### 3.4. Unfolding Test and In Vitro Drug Release Studies

**Figure 8:**
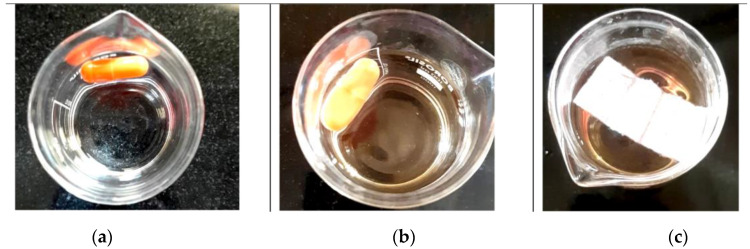
Unfolding test of optimized formulation filled in hard gelatin capsule. (**a**) Capsule added to the medium, (**b**) capsule started disintegration, and (**c**) fully unfolded film.

An unfolding test was conducted using HCL (pH 1.2) as the medium length. The capsules were broken down within 3–5 min. Formulated layers must unfold within 10–15 min after intake to avoid preterm eviction. The dimensions of the unfolded layer must be greater than the size of the relaxed pyloric sphincter [19]. The layers exhibited improved stickiness when in contact with the test medium. Various anti-adhesive agents were employed to avoid sticking (Figure 8).

**Figure 9:**
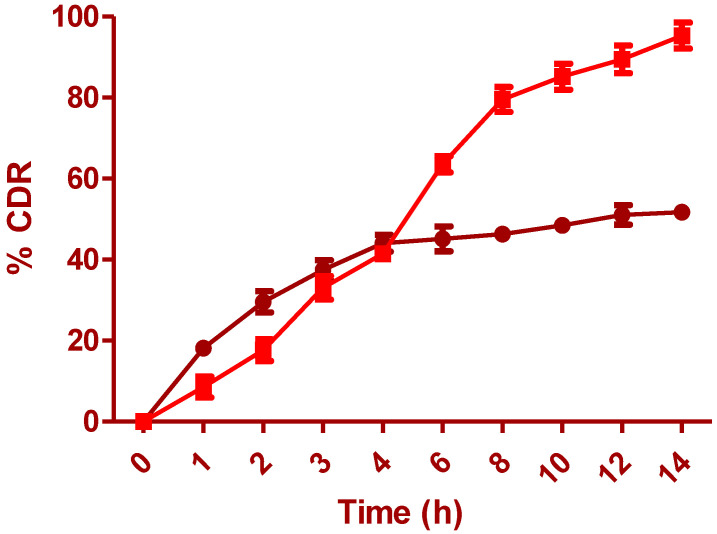
In vitro drug release comparison of pure IH and O-IH-UF formulation.

**Figure 10:**
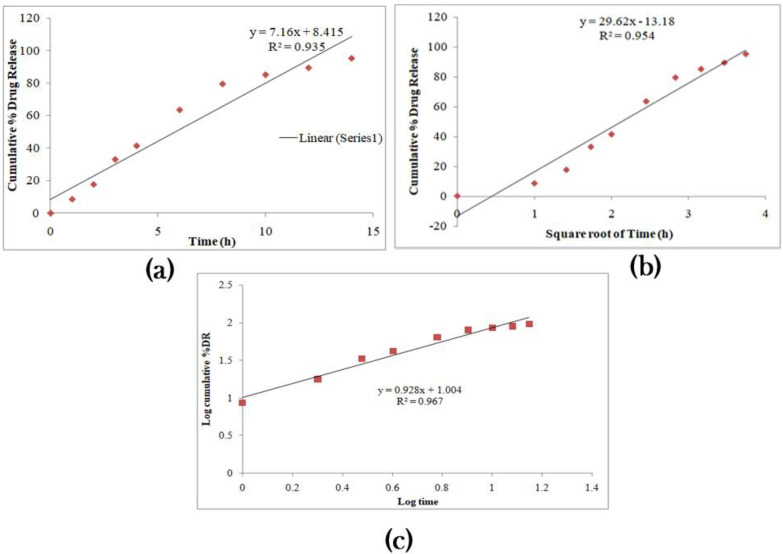
Drug [IH] release kinetics. (**a**) Zero-order kinetics, (**b**) Higuchi Plot, and (**c**) Korsmeyer–Peppas model from O-IH-UF.

IH was released from pure IH solution and the optimized formulation; results are shown in Figure 9. An improper IH release was seen in the dissolution of Pure-IH due to its solubility issues in the lower part of GIT. Quick-release of IH was seen in the first 3–4 h, and later a steady-state release was observed till the end of the study, as it reached the lower part of the GIT. The cumulative IH release from O-IH-UF showed a rapid release (3 h) of around 40–45% of IH from the total amount. The quick release at first is due to the presence of IH at the film’s surface, which allows a great diffusion of water through the liquid matrix, thus causing the quick release. Later, a steady phase with constant drug release is seen till 14 h. The total quantity of drugs released from standardized preparation was around 96.24%. This is attributed to the controlled action of hydrophobic Eudragit polymer. Eudragit polymer is water-insoluble and has very low permeability. Since this formulation did not contain any channeling agents, the formation of pores and cracks did not occur to facilitate drug release. Table 9 compares the difference and similarity factors between pure IH and O-IH-UF. These results conclude that the release profile of IH from optimized formulation was quite different (f1, 53.50 and f2, 28.63) from the pure IH release profile as per the acceptance criteria. Drug release kinetics of O-IH-UF are shown in Figure 10. IH release follows controlled release (r^2^ value of zero order kinetics, 0.9354) with an anomalous (Non-Fickian) diffusion mechanism. 

### 3.5. Stability Studies

There were no changes in the film’s physical appearance during the study course’s storage conditions. The dissolution profile of the test samples was compared by computing similarity and dissimilarity factors using the standardized formulation as a reference to ensure the drug release. All the stored samples exhibited a good similarity profile (>90) concerning the reference formulation (Table 10).

### 3.6. Buoyancy or Gastric Residence Studies of SPH

**Figure 11:**
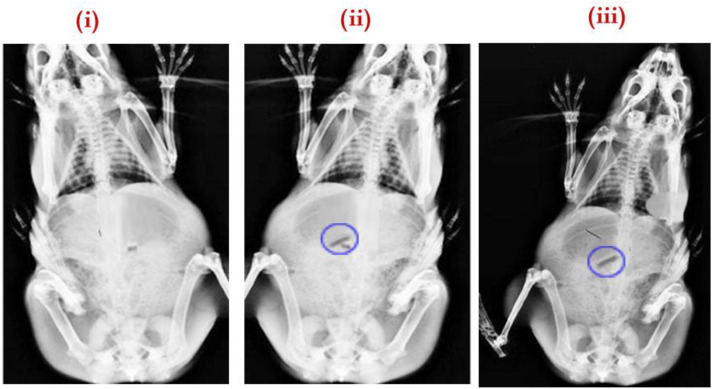
X-ray radiographs (**i**) before administration and after the administration of O-IH-UF at (**ii**) 1 h and (**iii**) 8 h.

The gastric residence behavior was observed in the rabbit stomach using the X-ray technique. An X-ray photograph was taken prior to the administration of the formulation (Figure 11i). The next X-ray photograph was taken after 1 h of administration (Figure 11ii). By this time, the capsule was disintegrated, and O-IH-UF was released into the stomach and remained buoyant. Obtained radiographic images at the end of 8 h revealed that the formulation had altered its position but remained buoyant in the stomach. The swelling of the SPH is visualized with a translucent swelling layer around it at the end of 8 h (Figure 11iii). Formulated unfolding film remaining buoyant in the stomach, and this study provides the required evidence that the unfolding film type was one of the ideal approaches to retain the dosage forms in the upper part of the GIT without transition to the lower part of the GIT. 

### 3.7. Pharmacokinetic Studies

**Figure 12:**
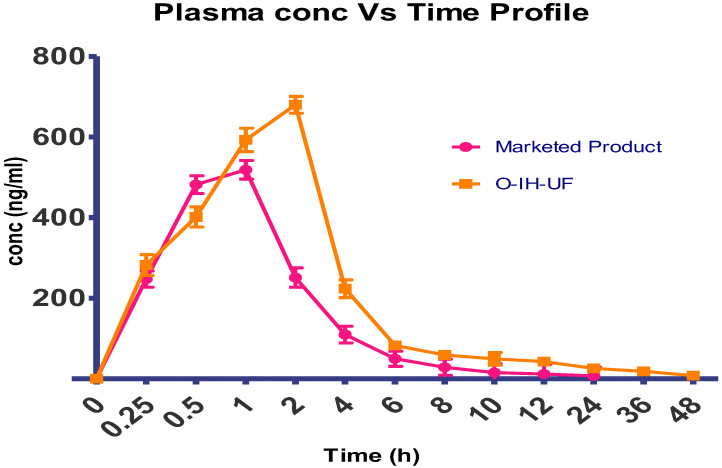
Pharmacokinetic profiles of marketed product and O-IH-UF.

From the data of plasma levels, the maximum plasma concentration (Cmax, ng/mL) and the time to achieve this concentration (Tmax, h) were calculated and reported as mean standard deviation (SD). The area under the curve from time 0 to time 24 was calculated using the trapezoidal rule (AUC0-24, ng/h/mL). Using PK-Solver, the elimination rate constant (Ke), half-life (t_1/2_), volume of distribution (Vd), and area under the curve from time 0 h to infinity (AUC0-α, ng/h/mL) were all determined. Table 11 and Figure 12 show the pharmacokinetic characteristics of the two samples.

The pharmacokinetic parameters in the two groups under study were compared. The marketed oral tablet was found to have a mean peak concentration of 518.56 ng/mL and a time to peak of 0.75 to 1 h. O-IH-UF, on the other hand, takes 2 h to reach the peaks and has mean peak concentrations of 679.78 ng/mL. The elimination rate for films that were unfolding was reduced by 2.14 times, and the MRT was extended to 7.54 h. The improved formula’s AUC [ng ml^−^^1^ h] was determined to be 2894.29, as opposed to the marketed formula’s [1570.10]. O-IH-UF had a relative bioavailability of 184.33 compared to commercially available products. A longer period was needed for the IH to dissolve before entering the intestines, which facilitated a better bioavailability. This demonstrates the usefulness of the unique drug delivery technique known as the projected unfolding film type in enhancing the therapeutic effectiveness of IH.

## 4. Conclusions

The study aimed to formulate an expandable delivery system that can prolong the release of IH for a minimum of 12 h and can be held in the GI system for a longer duration irrespective of fed/fasted conditions. This expansion results in improved bioavailability, reduced dose frequency, and side effects. In this study, a single-layered gastro retentive extendible film loaded with IH was prepared with the design of experiments. Execution of the optimized result achieves folding endurance of 115.29 and 25 of LTL. O-IH-UF was found to unfold within 15 min, ensuring preterm eviction avoidance. The developed formulation has shown considerable tensile strength, mechanical strength, and degradability. Young’s modulus test result was more significant than the set limit (0.302 N/mm^2^) and denoted high rigidity in the stomach. The in vitro drug release test confirms the complete and sustained release of IH by the end of 14 h. The floating and mechanical performance of the film revealed the gastro retentive potential of the dosage form. The kinetic studies of optimized formulation showed a significant rise in MRT and a decrease in the Ke, which is favorable for control release. This delivery system can be explored for further advancements through human trials that further confirm the enhanced bioavailability and good therapeutic outcomes of prevailing drugs of such type.

## Figures and Tables

**Table 1:** Experimental plan for Box-Behnken design in terms of actual and coded values. Three factors and three levels were considered to study their impact on folding endurance and LTL.

Factors/Independent Variables	Levels			Responses/Dependent Variables	Constraints
	−1	0	+1		
Concentration of Eudragit L 100-X_1_	50	100	150	Folding Endurance	Maximum
Concentration of PEG 400-X_2_	0.4	0.6	0.8	LTL	Maximum
Concentration of sodium bicarbonate-X_3_	0	10	20		

**Table 2:** Effect of anti-adhesive on the unfolding mechanism of IH film.

S. No	Anti-Adhesive Excipient	Unfolding	Length of Unfolded Film
1.	Microcrystalline cellulose	Not observed	-----
2.	Starch	Observed	9 mm
3.	Talc	Observed	11 mm
4.	Magnesium stearate, talc, and citric acid	Observed	11 mm
5.	Magnesium stearate and citric acid	Not observed	-----
6.	Citric acid, sodium bicarbonate, and talc	Observed	16 mm
7.	Citric acid and sodium bicarbonate	Observed	19 mm

**Table 3:** Experimental runs were conducted as per the BBD and their observed responses.

	Factor 1	Factor 2	Factor 3	Response 1	Response 2
Run	A:Eudragit L100	B:PEG 400	C:Sodium Bicarbonate	Folding Endurance	LTL
	mg	mL	mg		mm
13	50	0.6	0	76 ± 4	9 ± 1.5
11	50	0.6	20	82 ± 3	13 ± 2.1
14	50	0.4	10	69 ± 3	14 ± 2.3
2	50	0.8	10	87 ± 5	16 ± 2.4
12	100	0.4	0	74 ± 4	9 ± 2.6
1	100	0.4	20	74 ± 6	14 ± 1.8
16	100	0.8	0	97 ± 7	15 ± 2.2
15	100	0.6	10	84 ± 5	21 ± 2.5
7	100	0.6	10	86 ± 5	21 ± 2.4
8	100	0.8	20	108 ± 3	21 ± 2.0
9	100	0.6	10	84 ± 4	22 ± 0.7
3	100	0.6	10	85 ± 5	22 ± 1.8
17	100	0.6	10	86 ± 2	22 ± 1.6
6	150	0.6	0	86 ± 2	13 ± 1.3
4	150	0.4	10	76 ± 5	17 ± 1.4
5	150	0.6	20	98 ± 5	18 ± 1.5
10	150	0.8	10	114 ± 4	25 ± 1.6

**Table 4:** Model summary statistics of selected responses.

Responses	Source	Sequential *p*-Value	Lack of Fit *p*-Value	Adjusted R^2^	Predicted R^2^	
	Linear	<0.0001	0.0056	0.9031	0.8400	
	2FI	0.0020	0.0428	0.9695	0.9219	
	**Quadratic**	**0.0844**	**0.0832**	**0.9821**	**0.8997**	**Suggested**
	Cubic	0.0832		0.9931		Aliased
	Linear	0.0393	0.0004	0.3382	0.1322	
	2FI	0.9163	0.0002	0.1806	−0.5594	
	**Quadratic**	**<0.0001**	**0.1544**	**0.9756**	**0.8762**	**Suggested**
	Cubic	0.1544		0.9870		Aliased

**Table 5:** Model (quadratic) fit summary of the responses.

Parameter	PS	EE
Std. Dev.	1.61	0.7512
Mean	86.24	17.18
C.V. %	1.87	4.37
Adeq Precision	34.9242	28.5860
Lack of Fit F-value	4.75	3.06
Lack of Fit *p*-value	0.0832	0.1544
Model F value	98.74	72.17
Model *p*-value	<0.0001	<0.0001

**Table 6:** ANOVA coefficients table for both responses to confirm the significant factors for chosen responses. Bold terms indicate significance.

	Intercept	A	B	C	AB	AC	BC	A^2^	B^2^	C^2^
Folding endurance	85	7.5	14.125	3.625	5	1.5	2.75	−0.625	2.125	1.125
*p*-values		<0.0001	<0.0001	0.0004	0.0004	0.1055	0.0113	0.4531	0.0306	0.1959
LTL	21.6	2.625	2.875	2.5	1.5	0.25	0.25	−2.55	−1.05	−5.8
*p*-values		<0.0001	<0.0001	<0.0001	0.0052	0.5270	0.5270	0.0002	0.0241	<0.0001

**Table 7:** Relative error calculation for standardized preparation.

S. No	Response	Predicted/Theoretical Value	Experimental/Practical Value	Relative Error (%)	Limit for Relative Error (%)
1.	Folding endurance	115.293	117.00	−1.48	±5
2.	LTL	25.198	24.214	3.90	

**Table 8:** Evaluation tests for optimized formulation.

S. No	Formulation	Test	Result(Avg ± S.D)
1.	O-IH-UF	Weight variation	0.253 ± 0.023 g
2.		Thickness	0.93 ± 0.02 mm
3.		Mechanical strength	1.7 ± 0.03 kg/cm^2^
4.		Drug content	98.79 ± 0.10%
5.		Retention time	>10 h

**Table 9:** Comparison of difference and similarity factors between pure IH and O-IH-UF.

S. No	Reference	Test	Difference Factor (f1)	Acceptance Criteria	Similarity Factor (f2)	Acceptance Criteria
1.	Pure IH	O-IH-UF	53.50	0–15	28.63	50–100

**Table 10:** Stability studies for O-IH-UF.

TEST	INITIAL	25 °C ± 2 °C + 60% ± 5% RH		40 °C ± 2 °C + 75% ± 5% RH	
		3 M	6 M	3 M	6 M
Folding endurance	117	115	113	114	110
*f* 2	--	97.24	96.25	96.87	94.64

**Table 11:** Calculated pharmacokinetic parameters of marketed product and O-IH-UF.

S. No	Pharmacokinetic Parameter	Marketed Product	O-IH-UF
1.	C_max_ (ng mL^−1^ h)	518.56 ± 26.09	679.78 ± 19.24
2.	t _max_ (h)	1 ± 0	2 ± 0
3.	AUC_0-t_ (ng mL^−1^ h)	1570.10 ± 49.19	2894.29 ± 102.49
4.	AUC_0-i_ (ng mL^−1^ h)	1645.78 ± 35.26	3048.19 ± 139.73
5.	MRT_t_ (h)	4.21 ± 0.41	7.54 ± 0.53
6.	K_e_ (1/h)	0.1689 ± 0.019	0.0789 ± 0.0039
7.	Relative Bioavailability	---	184.33

## Data Availability

Data is contained within the article.
